# Congenital Nonprofound Bilateral Sensorineural Hearing Loss in Children: Comprehensive Characterization of Auditory Function and Hearing Aid Benefit

**DOI:** 10.3390/audiolres12050054

**Published:** 2022-10-07

**Authors:** Erik Berninger, Maria Drott, Mircea Romanitan, Lisbeth Tranebjærg, Sten Hellström

**Affiliations:** 1 Department of Clinical Science, Intervention and Technology, Division of Ear, Nose and Throat Diseases, Karolinska Institutet, 171 77 Stockholm, Sweden; 2 Department of Audiology and Neurotology, Karolinska University Hospital, 141 86 Stockholm, Sweden; 3 Department of Clinical Genetics, The University Hospital Rigshospital/The Kennedy Centre, DK-2600 Copenhagen, Denmark; 4 Institute of Clinical Medicine, University of Copenhagen, DK-1165 Copenhagen, Denmark

**Keywords:** children, congenital, electrophysiology, hearing, hearing aids, imaging, otoacoustic emissions, sensorineural hearing loss, speech recognition

## Abstract

A prospective cross-sectional design was used to characterize congenital bilateral sensorineural hearing loss (SNHL). The underlying material of >30,000 consecutively screened newborns comprised 11 subjects with nonprofound, alleged nonsyndromic, SNHL. Comprehensive audiological testing was performed at ≈11 years of age. Results showed symmetrical sigmoid-like median pure-tone thresholds (PTTs) reaching 50–60 dB HL. The congenital SNHL revealed recruitment, increased upward spread of masking, distortion product otoacoustic emission (DPOAE) dependent on PTT (≤60 dB HL), reduced auditory brainstem response (ABR) amplitude, and normal magnetic resonance imaging. Unaided recognition of speech in spatially separate competing speech (SCS) deteriorated with increasing uncomfortable loudness level (UCL), plausibly linked to reduced afferent signals. Most subjects demonstrated hearing aid (HA) benefit in a demanding laboratory listening situation. Questionnaires revealed HA benefit in real-world listening situations. This functional characterization should be important for the outline of clinical guidelines. The distinct relationship between DPOAE and PTT, up to the theoretical limit of cochlear amplification, and the low ABR amplitude remain to be elucidated. The significant relation between UCL and SCS has implications for HA-fitting. The fitting of HAs based on causes, mechanisms, and functional characterization of the SNHL may be an individualized intervention approach and deserves future research.

## 1. Introduction

The introduction of universal newborn hearing-screening (UNHS) has changed pediatric audiology substantially—like a “silent revolution” [1]. While the UNHS [2,3,4] and early intervention with hearing aids (HAs) seem to be beneficial (e.g., [5,6,7]), and cost-effective [3,8], early diagnostics and intervention are indeed challenging. The current lack of comprehensive auditory characterization of typical congenital sensorineural hearing loss (SNHL) hampers further advances in early intervention. More knowledge is needed on the causes (see recent reviews on etiological evaluation [9,10]), mechanisms, and the resulting dysfunction, to enhance subsequent communicative capacity.

The long-term goal is to find the causes and mechanisms behind various nonsyndromic congenital SNHLs and to identify or develop future treatment options. Here, we try to characterize the summary auditory profile and potential mechanisms of alleged nonsyndromic, nonprofound, bilateral congenital SNHL. A comprehensive audiological test battery along with magnetic resonance imaging (MRI) was used. The study group consisted of subjects with pure nonprofound SNHL, as identified at birth (part of [11]). They were 9–13 years old at the time of inclusion. The reason for choosing this study group, recruited from [11], was the well-defined material of consecutively identified SNHLs at birth, no problem with acoustical effects due to growth of the outer ear canal [12] or neural maturation in a frequency-dependent way [13,14] as in young children, and the opportunity to use advanced psychoacoustical tests and questionnaires.

The study group should represent a clinically relevant and representative sample of congenital alleged nonsyndromic, nonprofound, bilateral SNHL, as our UNHS, which is based on multiple recordings of transient evoked otoacoustic emissions (TEOAEs) and clinical auditory brainstem response (ABR), is highly efficient [11,15,16]. High coverage rate (98%), a high proportion of identified congenital SNHL (0.17% bilateral; 0.06% unilateral), and ABRs for site-of-lesion testing and threshold determination down to 20 dB nHL have been the basis for early characterization of type and degree of hearing impairment (HI). Our entire material of subjects with congenital HI is based on >30,000 consecutively screened newborns during a 6-year period [11].

Most SNHLs are localized within the cochlea thereby disturbing its delicate function. The healthy cochlea comprises the micromechanically active amplification system, termed the cochlear amplifier [17], which increases the maximum sensitivity of the ear by 40–60 dB [18]. The cochlear amplifier is also essential for high-frequency selectivity, compressive nonlinearity, and various forms of OAEs, i.e., cardinal features for excellence in hearing (e.g., [19]).

This prospective cross-sectional study aimed to characterize the summary auditory profile, function, and potential mechanisms of congenital, alleged nonsyndromic, nonprofound, bilateral SNHL. A comprehensive test battery was used at ≈11 years of age. Specifically, cochlear morphology, auditory nerve morphology, cochlear function, and central auditory function were studied in relationship with unaided and aided (HAs) recognition of speech in spatially separate competing speech (SCS) and questionnaires reflecting problems in real-world listening situations.

Detailed characteristics of the auditory function will be reported. Dysfunctions at anatomical structures will be discussed along with the functional benefits of early HA-intervention.

## 2. Materials and Methods

### 2.1. Subjects

#### 2.1.1. Inclusion Criteria

All subjects with (alleged) nonsyndromic bilateral cochlear hearing loss exceeding ≈30 dB HL (i.e., ABR threshold (ABRthr) >20 dB nHL) who participated in the 10-year assessment of a 6-year UNHS ([11], n = 52 bilateral SNHL). They were healthy according to a standard medical protocol.

#### 2.1.2. Exclusion Criteria

Any syndromic disease (Down’s, Lennaux Gastaut, Treacher-Collins, Long-QT, Kabuki, Lange-Nielsen, Monosomy 18q, and Hurler as stated in the medical files; n = 9 (2 Down’s)), conductive hearing loss, cleft palate, cerebral palsy (n = 4), epilepsy, and asphyxia (see Table I in [11]). Severe to profound bilateral SNHL (i.e., no measurable hearing thresholds as assessed 10 years after start of the 6-year UNHS, or, subjects using at least one cochlear implant (CI); n = 18). Abnormal ABR I-V latency at birth. (Numbers are given for the most common reasons for exclusion.)

#### 2.1.3. Eligible Subjects

In all, 18 subjects, aged 9–13, fulfilled the inclusion and exclusion criteria, i.e., subjects no. 1, 2, 5, 6, 8, 10, 11, 14, 16, 26, 27, 28, 34, 37, 38, 42, 43, and 49 according to Table I in [11] (see Appendix A for detailed characteristics of eligible subjects and reasons for not participating in the study). All but one of the 18 subjects (no. 10 [11]) were fitted with, and used, HAs in both ears.

#### 2.1.4. Participating Subjects

Two of the 18 eligible subjects for this study had attention deficit hyperactivity disorder (ADHD), or, pending ADHD, 2 moved, and 3 were not interested in participating. Hence, the 11 participating children should be a representative sample for alleged nonsyndromic nonprofound congenital SNHL. The term “alleged nonsyndromic” is used as the medical files did not reveal any syndromic disease (that only can be ruled out entirely by comprehensive genetic testing (e.g., [20,21])) for the 11 otherwise healthy participating subjects, see Table I in [11]. Seven males (subjects no. 1, 4, 5, 7, 8, 9, 11) and 4 females (2, 3, 6, 10, according to testing order) participated in this study (i.e., 64% males). Detailed characteristics for the 11 participating subjects, as obtained at birth and in an earlier follow-up, can be found in Table I in [11]. Subject no. in this study vs. the corresponding subject no. in Table I [11] are as follows: 1 (14), 2 (8), 3 (37), 4 (16), 5 (10), 6 (38), 7 (6), 8 (43), 9 (11), 10 (49), and 11 (26). Median age at test was 11.0 years (9–13 years).

Median age for the 11 participating subjects at conclusive ABRthr was 1.7 months (1.0–10.5 months; QR 1.5–4.0 months). The degree of SNHL vs. frequency was obtained at a median age of 60 months (27–83 months; QR 49–74 months). Median ABRthr at birth for the left and right ears was 50 dB nHL (35 dB nHL—no response; QR 40–60 dB nHL), and 45 dB nHL (35 dB nHL—no response; QR 40–50 dB nHL), respectively.

### 2.2. Study Design

Immediately before the hearing tests, the subjects underwent otomicroscopic examination, tympanometry, and acoustic stapedius reflex measurements to ensure normal middle ear conditions. All the subjects underwent recordings of pure-tone threshold (PTT) and uncomfortable loudness level for pure tones (UCL) in both left and right ears (randomized testing order across subjects), but most of the audiological tests were only performed in the better ear (except sound field measurements). The better ear was defined as the ear with the lowest pure-tone average (PTA, 500–2000 Hz). All the recordings were performed in one audiometric test room (double-walled soundproof booth) with low ambient sound levels allowing PTT determinations down to −10 dB HL [22]. All the monaural measurements were performed via one set of insert earphones (Ear Tone ABR, Etymotic Research, Inc, Elk Grove Village, IL, USA). For details regarding the study day, please see Appendix A.

MRI was performed after the study day.

### 2.3. Stimulus and Recording Equipment

The stimulus and recording system consisted of a personal computer, Tucker Davis Technologies (TDT; Alachua, FL) system 3 signal processing unit RP 2.1 including sound amplification unit, HB7 (TDT; −27 dB gain) for all measurements except sound-field recordings for which a sound card (Echo Audiofire 12, Echo Audio Corporation, Carpenteria, CA, USA) connected to the personal computer was used.

The TDT signal processing unit was used for sound presentation, data acquisition, and signal processing. The software was written in Matlab™ (The Math-Works Inc., Natick, MA, USA).

### 2.4. Hearing Dynamic Range for Pure Tones

#### 2.4.1. Pure-Tone Threshold (PTT)

PTTs were measured monaurally at 125, 250, 500, 1000, 2000, 3000, 4000, 6000, and 8000 Hz using a computerized fixed-frequency Békésy technique, with four turning-point pairs [23], according to [24,25]. The first tested ear (L/R) was chosen at random (based on the national ID number). The subjects had one training session (at 1000 Hz) before the PTT recording.

The stimulus consisted of pulsating tones (400 ms pulse duration, 50% duty cycle) presented at a level change rate of 3 dB/s (steps of 1.25 dB). (See Appendix A for further details on PTT recording and determination of PTT reliability).

According to a hypothesis proposed by McFadden [26], the effects of ear and sex might be due to prenatal masculinization processes and asymmetrical behavior of the efferent medial olivocochlear system. Hence, ear asymmetries and sex effects of the precisely recorded PTTs were studied in this group of congenital SNHL. We have earlier demonstrated statistically significant differences in neonatal TEOAE-levels [15] and TEOAE pass ratios [11]. A predominance of males and left ears (congenital unilateral SNHL) has been observed as well [11].

#### 2.4.2. Uncomfortable Loudness Level for Pure Tones (UCL)

UCL was measured monaurally according to the Swedish Audiometry Methods Group [27], at 500, 1000, 2000, 3000, 4000, and 6000 Hz, in steps of 5 dB, using the TDT 3 signal processing unit RP 2.1 (TDT, Alachua, FL, USA) and custom-made software. The start ear (L/R) was the same as for PTT. The maximum UCL tested was 110 dB HL. Each subject received verbal and written information on the test procedure from the research audiologist using exactly the wording stated in the Swedish clinical guideline [27].

### 2.5. Otoacoustic Emissions (OAEs)

The pre-neural nature of OAEs (e.g., [28]) offers a non-invasive measure of cochlear function. The motile outer hair cells (OHCs) within the organ of Corti are key elements for the generation of OAEs, which are epiphenomena of the active, nonlinear, amplification process. Clinically, the most common forms of OAEs are TEOAEs and distortion product otoacoustic emissions (DPOAEs). Various cochlear dysfunctions could be studied by OAEs; for example, the TEOAEs for hair cell integrity and the DPOAEs for the function of horizontal stereocilia connections [29]. According to the mammalian taxonomy proposed by Shera and Guinan [30], the TEOAEs evoked at low stimulus levels are generated by coherent reflection at the peak of the traveling wave (see e.g., [31]), whereas the DPOAEs are generated by non-linear distortion and linear reflection at the 2f1−f2 tonotopic place. The TEOAEs are temporally shifted from the stimulus and show an overall nonlinear growth rate of approximately 0.4 dB/dB below saturation [32]. The DPOAEs are spectrally shifted from the two continuous primary tones (f1 < f2; e.g., f2/f1 = 1.22). The growth rate is 0.7–0.8 dB/dB [33], though high variability in slope and shape exists [34]. The nonlinear interaction at frequencies close to f2 is the most important frequency region for generating the DPOAE.

The most prominent distortion product (2f1-f2) is 60 dB below the equal-level primaries in humans (e.g., [35]). This DPOAE can be audible down to primary tone levels 15–20 dB above the hearing threshold [36].

Although the stimuli are different for TEOAE and DPOAE, transient and two continuous tones, respectively, both responses are frequency specific to cochlear function (see [32]). Signal-to-noise ratio (SNR) was used as a reasonable measure for comparison of the two various forms of OAEs, i.e., the band-pass filtered TEOAE SNR and DPOAE SNR, due to e.g., differential analysis bandwidths (band-pass and narrowband filter, respectively). Each signal and noise component was analyzed in a similar way and SNR is thus appropriate when comparing the two forms of OAEs.

#### 2.5.1. Transient Evoked Otoacoustic Emission (TEOAE)

TEOAE was recorded in the non-linear mode with ILO 292 (Otodynamics Ltd., UK) using an analysis time window of 6.0–20.5 ms (260 sweeps). In order to increase the sensitivity for detection of weak TEOAEs, i.e., presumably when the TEOAE detection threshold and slope of the I/O function are increased, the stimulus level was chosen as high as possible (see earlier human-experimental studies for details [37,38,39]). The used stimulus peak equivalent sound pressure level was 87 dB SPLp.e., as recorded in the ear canal. The response level for the entire response (RMS) as well as for band-pass filtered (1/2 octave) TEOAE (TEOAEb.p.) was analyzed.

#### 2.5.2. Distortion Product Otoacoustic Emission (DPOAE)

DPOAE was measured as the 2f1−f2 cubic distortion product component, using ILO 292 (Otodynamics Ltd., UK). The frequency ratio of the primaries was 1.22 (f2/f1) and the stimulus consisted of equal-level sinusoids at 75 dB SPL, i.e., the expected sound pressure level at the tympanic membrane. This stimulus has been used successfully for the study of reversible, concentration-dependent, quinine-induced cochlear hearing loss at the level of the OHCs in normal-hearing (NH) volunteers [40]. The DPOAE was recorded with f2 at 1000, 1500, 2000, 3000, 4000, and 6000 Hz. The DPOAE signal-to-noise ratio (SNR) was expressed as DPOAE level − (Noise + 2 SD). The intermodulation distortion (2f1-f2) in the DPOAE instrument was low, <−20 dB SPL (1000–6000 Hz), as recorded at 75/75 dB SPL in the Otodynamics probe test cavity (A80-14-1-0, Otodynamics Ltd., UK) in an audiometric test room.

As the micromechanical amplification system within the cochlea increases the sensitivity of the ear by 40-60 dB [18], post-hoc analysis comprised DPOAE as a function of PTT for PTT ≤ 60 dB HL, i.e., for PTTs where we expected to find DPOAEs. Linear mixed modeling of DPOAE level with PTT and frequency as fixed effects was performed (Satterwaite’s method for *t*-tests).

### 2.6. Psychophysical Tuning Curve (PTC)

Frequency selectivity, which is highly dependent on the function of the OHCs, was measured at 2000 Hz, using PTC according to [41,42]. PTC could be used to study e.g., the upward spread of masking and the tail resembling the function of the inner hair cells (IHCs). The PTC should reflect the cochlear tuning (see e.g., [43]) and should not be dependent on age per se.

The probe was presented 10 dB above the PTT at 2000 Hz, while the masker (dB HL) was presented at 860, 1560, 1840, 2000, 2160, 2460, and 2960 Hz. The probe was a pulsed sinusoidal with on-period 200 ms (50% duty cycle). The onset and offset of the probe were cosine shaped. The masker was a narrowband noise (10 dB relative bandwidth, 0.25).

### 2.7. Wideband Acoustic Stapedius Reflex Threshold (WBART)

WB Tympanometry and WBART [44,45] were recorded with the Interacoustics WB tympanometry research instrument (software release 3.2.1, Interacoustics, Denmark) for functional studies of the IHCs and the reflex arc. The WBART was elicited ipsilaterally, at tympanic peak pressure [46], at the stimulus frequencies of 1000, 2000, and 4000 Hz, using an ascending method in steps of 5 dB. The maximum tonal stimulus level was 105 dB SPL (as recorded in a 2-cc coupler). The WBART threshold was defined as the low-frequency threshold (AR-L).

RETSPL was 6.5, 12.0, and 3.5 dB SPL at 1000, 2000, and 4000 Hz, respectively (Interacoustics 2cc-coupler, Interacoustics standard).

### 2.8. Click-Evoked ABR

While the ABR is used mainly for site-of-lesion testing and threshold estimation, the ABR input/output (I/O) function [23] could be used to estimate the dynamic range of hearing for the fitting of nonlinear HAs to newborns. The ABR can also reveal important diagnostic information, for example, for the determination of loudness recruitment [39,47], cochlear synaptopathy [48,49], and carboplatin-induced hearing loss [50].

ABRs were recorded using 100 µs rarefaction clicks in 10 dB steps, from 80 dB nHL [51] down to threshold in steps of 10 dB by use of EP 15 (Interacoustics, Denmark), as pre- and isolation amplifier (100 dB gain (including a custom-made built-in additional amplifier with 20 dB gain), 7–8000 Hz), connected to TDT 3 signal processing unit RP 2.1 (Tucker Davis Technologies, Alachua, FL, USA). Repetition rate, 39 Hz (cosine-tapered analysis time window, 15 ms; 0–100% within 0.75 ms; sampling frequency, 48.8 kHz). The instrumentation was the same as in [23]. Calibration of 100 µsec clicks was performed according to [51] in an occluded ear simulator [24], i.e., 35.5 peRETSPL in dB (peak-to-peak equivalent reference threshold SPL).

Ag/AgCl electrodes were placed on left and right mastoids (left, right), and the forehead (vertex, ground), for differential recording between the ipsilateral mastoid (noninverting) and the forehead (inverting). Lights in the audiometric test room were turned off during the ABR recordings to minimize electric interference. The response quality was enhanced by the use of up to 10,000 sweeps at stimulus levels close to the ABR threshold, i.e., the number of sweeps was increased with decreasing stimulus levels in order to increase the SNR. Each sweep was digitally filtered (30–8000 Hz, Butterworth filter; 2-pole low pass and 2-pole high pass) and stored in a data file for off-line analysis.

The normalized cross-correlation coefficient between interleaved responses (ρ) was calculated in the time domain for the entire nonfiltered ABR (see [23]). The ABRthr (i.e., ABRthr (0.70)) was defined as the lowest stimulus level at which ρ ≥ 0.70 (ρ = 0.70 corresponds to SNR = 3.7 dB [23]). The amplitude (RMS) for the entire nonfiltered ABR (at ρ = 0.70; i.e., the number of consecutive sweeps needed to reach ρ = 0.70 (off-line analysis), see [23]) could be compared with our previously obtained material on young, healthy, normal-hearing adults aged 18–35 (n = 14; 50% females) at ρ = 0.70 [23]. The comparison of amplitudes is thus independent of the number of sweeps.

The comparison of ABR RMS amplitudes between the children with SNHL and the NH adults could be performed as both materials were obtained by using identical stimulus- and analysis paradigms [23] and children around 11 years of age show adult-like click-evoked ABRs. For example, wave-I latency is mature by 45–50 week conceptional age [52], whereas age-dependent effects on latencies for waves III and V are completed at 2–3 years of age [52,53]. Amplitude-intensity [54] and loudness functions [55] for click-ABRs are similar for NH children and adults. Moreover, there is no change in composite click-evoked ABR amplitude (data collapsed over peaks I to VI) between 5 years of age and adulthood [56].

ABR I, III, and V amplitude and ABR wave I-V latency (total number of sweeps) were measured at 80 dB nHL to allow comparison with NH adults (filter setting 300–1500 Hz, i.e., the amplitude could be measured between a well-defined vertex-positive maximum and following minimum).

It has been shown that the ABR wave-I suprathreshold amplitude could be used to reflect loudness recruitment [47]. Despite distinctly elevated thresholds, the wave-I amplitude is normal at high stimulus levels [47]. For a selective, concentration-dependent, flat-frequency, and reversible quinine-induced hearing loss at the level of the OHCs (NH volunteer), the wave V-amplitude does not change at stimulus levels ≥ 60 dB nHL, whereas the amplitudes are distinctly reduced at 40–50 dB nHL for a reversible PTT shift (1000–4000 Hz) of 34 dB [39]. The latency/stimulus-function showed only a slight offset, 0.2 ms [39]. Hence, suprathreshold ABR amplitudes are relevant for diagnostic purposes.

Data acquisition time zero was defined as stimulus onset in a Brüel & Kjær 4157 occluded ear simulator [24].

### 2.9. Recognition of Speech in Spatially Separate Competing Speech

Unaided and aided SCS was recorded at frontal incidence in an audiometric test room (quasifree sound field, 5 loudspeakers in total, adaptive method, threshold corresponded to 40% recognition of speech in 63 dB SPLCeq non-correlated and spatially separate competing speech, for details see [57]). The testing order between unaided and aided tests was randomized (on the basis of the national ID number).

Subjects were told to adjust their non-linear HAs to the mode normally used for speech. All the HAs were fitted on the basis of Desired Sensation Level version 5.0 [58].

Regression analysis was planned and performed for unaided SCS vs. UCL in the better ear at 2000 Hz, to study if elevated UCL (i.e., plausible reduction of afferent signals) was linked to deterioration in SCS. The 2000 Hz was chosen as an important frequency for recognition of speech (see [59] for importance functions).

### 2.10. Aided Frequency-Modulated Tone Threshold in Sound Field

Aided frequency-modulated tone threshold (FMT) in a quasifree sound field was recorded at frontal incidence in the audiometric test room immediately after the SCS (i.e., the HA parameters were the same as during the speech recognition test), to study audibility for soft sounds. Pulsing FM-tones were presented at the centre frequencies of 500, 1000, 2000, 3000, 4000, and 6000 Hz (+/− 12.5% frequency-deviation, 20 Hz modulation frequency, amplitude variation ≈ 1 dB/s, pulse duration = 400 ms, 50% duty cycle). The hearing thresholds were recorded with a computerized Békésy-technique, always starting at 30 dB HL [60]. The FMT was computed as the mean (in dB) of four turning-point pairs (see [23,39]). The subjects who used HAs with (active) gain controls were told not to change the HA gain after the SCS test.

### 2.11. Questionnaires

The abbreviated profile of hearing aid benefit (APHAB) [61,62] was used to quantify hearing disability in real-world listening situations. The APHAB questionnaire consists of 24 items divided into 4 listening categories. Unaided and aided percent of problems were calculated for each listening category (allowing comparison with, e.g., adult first-time HA users, n = 200; [63]). Valid data for each subject was based on n ≥ 4 answers (of 6) in each listening category (i.e., ease of communication (EC), reverberation (RV), background noise (BN), and aversiveness of sounds (AV)) in both the unaided and the aided listening situations, thereby allowing calculation of aided benefit (in the same way as in [63]).

### 2.12. Imaging

MRI was used to study the morphology of the inner ears and the vestibulocochlear nerves (cochlear morphology, 15 min investigation time, in all 30 min). Specifically, MRI could be used to diagnose congenital anomalies, as well as, infectious, inflammatory, or tumor pathology. Pathological changes of the cochlear and vestibular nerves in the internal auditory canal (IAC) and malformations that involve both the osseous and the membranous labyrinth (malformed otic capsules) are important etiological factors for SNHL.

MRI was performed on a 3T Siemens MRI Scanner. The sequences consisted of:

- Transversal high-resolution 3D T2-weighted images over IAC, inner ear, reconstructed in coronar and oblique sagittal plane.
- Coronar 3D FLAIR over brain including IAC, inner ear, reconstructed in coronar and oblique sagittal plane.
- Transversal high resolution 2D T2-weighted fast spin-echo images over brain, IAC.
- Transversal high resolution 2D T1-weighted fast spin-echo images over brain, IAC.

### 2.13. Statistical Analysis

All the statistical analysis was performed with Statistica version 13 (Statsoft Inc., Tulsa, OK, USA).

The nonparametric Wilcoxon matched pairs test was used to study lateral asymmetries in PTA and aided differences in SCS and questionnaires. Main effects ANOVA with frequency and subject as categorical variables was used to study lateral asymmetries in paired PTTs.

The nonparametric Mann–Whitney U test was used to study the effects of sex on PTA, differences between children and adults for ABR RMS and conventional wave-amplitudes, and unaided SCS for normal and elevated UCLs.

Kolmogorov–Smirnov test was used to compare cumulative distributions of ABR RMS amplitudes in hearing-impaired children and NH adults.

Regression analyses were used to study relationships between PTT in the worse vs. better ear; UCL vs. PTT; TEOAEb.p. SNR vs. DPOAE SNR; DPOAE level (and SNR) vs. PTT; SCS vs. UCL and PTT; PTT vs. ABRthr; ABR I-V latency vs. age at HA fit; APHAB vs. PTA, FMT average, and HA usage time.

## 3. Results

### 3.1. Hearing Dynamic Range for Pure Tones

#### 3.1.1. Pure-Tone Threshold (PTT)

**Figure 1:**
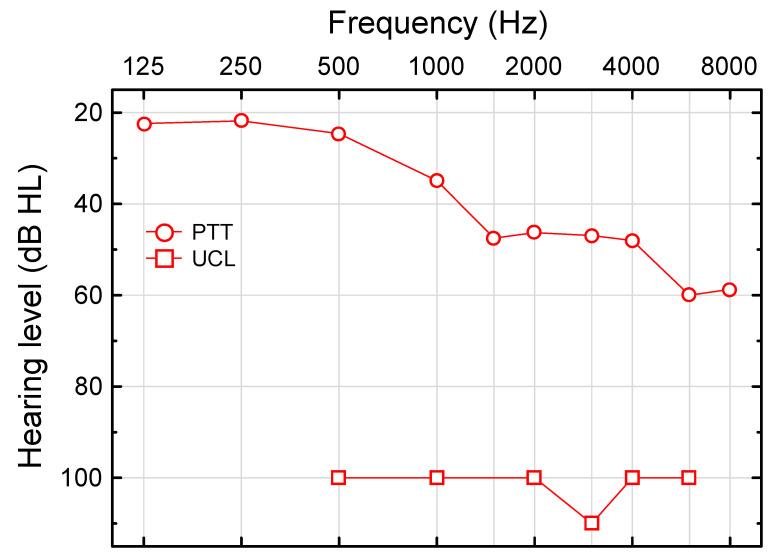
Median pure-tone threshold (PTT) and median uncomfortable level for pure tones (UCL) as a function of frequency in the better ear (n = 11 at all the frequencies, except for PTT at 8000 Hz, n = 10). The typical hearing dynamic range decreased from 75 dB at 500 Hz to 40 dB at 6000 Hz.

**Figure 2:**
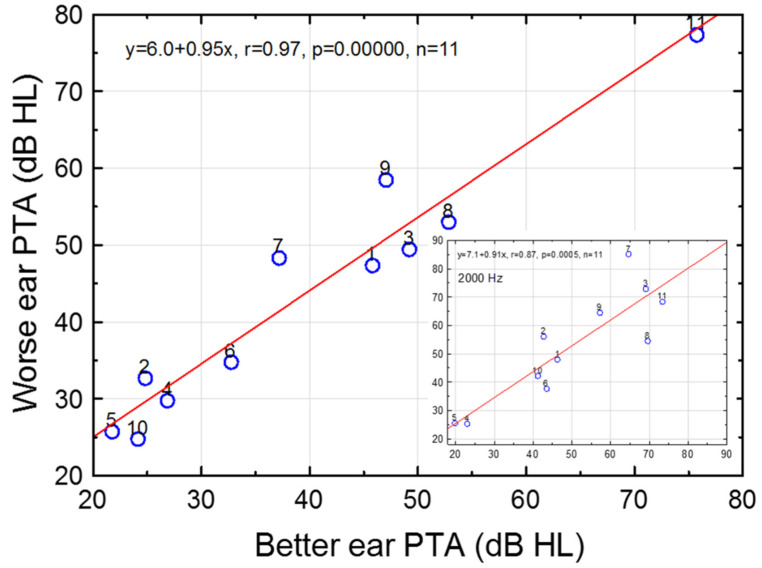
Pure-tone average (PTA, 500-2000 Hz) in worse versus better ear for all the participating subjects (1–11, numbered in testing order). Linear regression revealed worse ear PTA (dB HL) = 6.0 + 0.95∙better ear PTA (dB HL), r = 0.97, *p* = 0.00000 (n = 11). Inset depicts worse ear versus better ear pure-tone threshold at 2000 Hz (dB HL, n = 11). Note that 4 subjects had PTAs <30 dB HL in the better ear (2 subjects revealed PTTs <30 dB HL at 2000 Hz), thus reflecting the highly efficient UNHS.

On a group level, PTT as a function of frequency resembled a symmetrical sigmoid function (see Table S1). Median PTTs in the better ear increased from 20–30 dB HL at lower frequencies to 50–60 dB HL at ≥1500 Hz (Figure 1). The scatter in hearing thresholds for worse and better ears is depicted in Figure 2. The mean (SD) PTA in the worse and better ears was 43.9 (16.1; 24.8–77.5) dB HL and 39.8 (16.3; 21.7–75.7), respectively (n = 11). Quite symmetrical hearing thresholds at large were reflected by a high correlation between worse and better ear PTAs (r = 0.97, *p* = 0.0000, n = 11); the slope and intercept of the regression line were 0.95 and 6.0 dB HL, respectively (Figure 2 and Inset in Figure 2 for PTT at 2000 Hz).

Recordings of the PTTs revealed high reliability; SD for a single PTT was 0.7 and 0.9 dB in the left and right ear, respectively (1000 Hz, n = 11).

#### 3.1.2. Uncomfortable Loudness Level for Pure Tones (UCL)

Median UCLs in the left, right, better, and worse ears were all around 100 dB HL (500–6000 Hz), i.e., around the normal UCL across frequencies [64]. For example, the median UCLs in the better ear were 100 dB HL, except at 3000 Hz (Figure 1). The worse ear revealed median UCLs of 100, 95, 100, 95, 110, and 105 dB HL at 500, 1000, 2000, 3000, 4000, and 6000, respectively (n = 11 at all the frequencies).

The similarity to normal UCLs (see [64]), along with median PTTs of 46–60 dB HL (≥2000 Hz, Table S1), reflected the recruitment of loudness for pure tones. The typical hearing dynamic range in the better ear decreased from 75 to 40 dB at 500–6000 Hz (Figure 1).

### 3.2. Effects of Ear and Sex on Pure-Tone Thresholds

A minor systematic effect of ear on PTTs (paired differences) was found (F(1,88) = 8.2, *p* = 0.005). No effect of subject (F(10,88) = 0.77, *p* = 0.66), nor frequency (F(9,88) = 0.97, *p* = 0.47), existed for the paired differences. The left-right PTT difference averaged 2.6 dB (n = 108, all frequencies), i.e., worse PTTs in left than right ears. In addition, the mean left-right ear PTA difference, 3.6 dB (SD = 4.8 dB, −1.8–11.6 dB, n = 11), was also significantly different from zero (Wilcoxon matched pairs test, *p* = 0.04, n = 11). The PTA averaged 43.6 and 40.0 dB HL for the left and right ears, respectively.

No statistically significant effect of sex was found in the left or right ear PTA (*p*’s > 0.22, Mann–Whitney U test).

### 3.3. Otoacoustic Emissions (OAEs)

**Figure 3:**
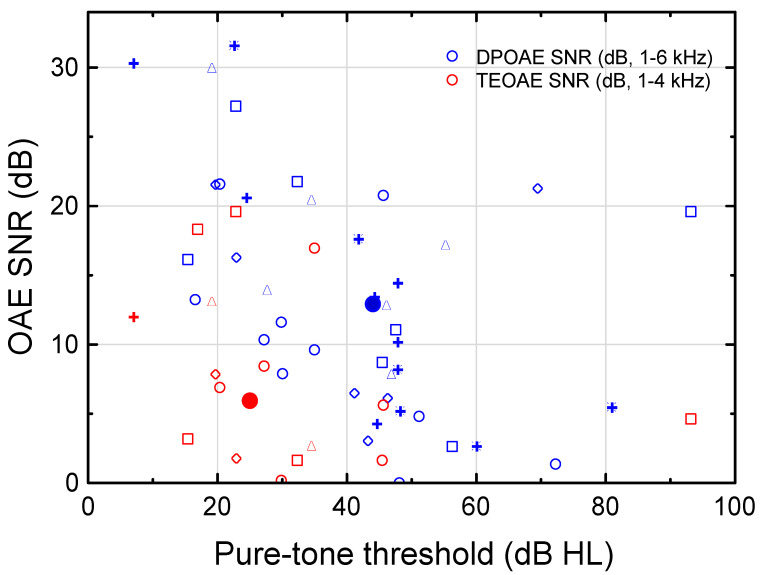
Bandpass-filtered transient evoked otoacoustic emission (TEOAE) SNR (red symbols, 1000–4000 Hz center frequency) evoked at 87 dB p.e. SPL and 2f1-f2 distortion product otoacoustic emission (DPOAE) SNR (blue symbols, 1000–6000 Hz) measured with equal-level sinusoids at 75 dB SPL as a function of pure-tone threshold (PTT). All OAE SNRs ≥ 0 dB. Large red and blue filled circles denote median OAE SNR at median PTT across frequencies for TEOAE and DPOAE, respectively. ○, □, ◇, △, **+**, and 🞼 denote 1000, 1500, 2000, 3000, 4000, and 6000 Hz, respectively. (11 subjects, 6(5) DP(TE) OAE frequencies.)

Distinct dissimilarities were found between DPOAEs and TEOAEs. Approximately half of the recorded DPOAEs exhibited positive SNRs in comparison with a minority of the TEOAEs (Figure 3). The DPOAEs existed in subjects with relatively worse hearing thresholds. In order to compare the TEOAEb.p. (1–4 kHz) and the DPOAE (1–6 kHz) exhibiting SNR ≥ 0 dB, the median (PTT, TEOAEb.p.SNR) and median (PTT, DPOAE SNR) was computed as (25 dB HL, 6 dB; n = 16), and (44 dB HL, 13 dB; n = 41), respectively (see large red and blue filled circles in Figure 3). Consequently, the DPOAEs could typically be measured for 19 dB worse SNHLs than the TEOAEs. No statistically significant relationship was demonstrated between TEOAEb.p. SNR and DPOAE SNR (regression analysis; SNR was used as a reasonable measure for comparison of the two different forms of OAEs). In all, Figure 3 demonstrates that important clinical information on cochlear function for mild-to-moderate SNHL could be obtained when using various OAEs evoked at high stimulus levels (cf. [39,40]). The DPOAE broadens the range of SNHL for which the cochlear function (e.g., compressive nonlinearity) could be analyzed. It may be noted that SNRs are well above zero in UNHS to guarantee a high enough OAE level for normal hearing, but here we analyzed OAEs in subjects even with slight SNHL and thereby low OAE SNRs.

**Figure 4:**
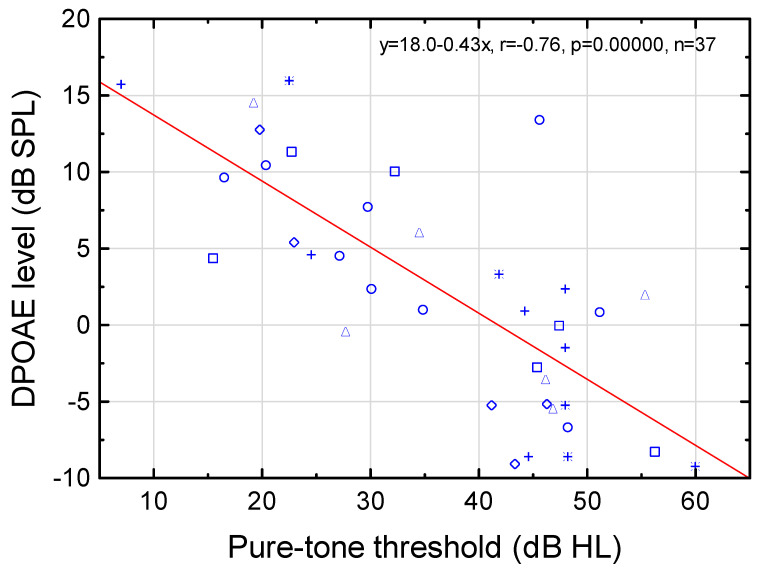
2f1-f2 distortion product otoacoustic emission (DPOAE) level as a function of pure-tone threshold (PTT) at 1000–6000 Hz (PTT ≤ 60 dB HL). Linear regression analysis revealed DPOAE level (dB SPL) = 18.0–0.43∙PTT (dB HL), r = −0.76, *p* = 0.00000, n = 37. ○, □, ◇, △, **+**, and 🞼 denote 1000, 1500, 2000, 3000, 4000, and 6000 Hz, respectively (n = 9, 6, 5, 6, 6, and 5, respectively). See regression equations for DPOAE vs. PTT at the various f2 frequencies in Table 1.

DPOAE decreased with increasing PTT (≤60 dB HL) (Figure 4). At frequencies ≥1500 Hz, the slopes of the regression lines for the DPOAE level vs. the PTT were significantly different from zero, except at 3000 Hz (similar to DPOAE SNR) (Table 1). Linear mixed modelling revealed a highly significant effect of PTT on the DPOAE level (r = −0.82, *p* = 1∙10^−6^), but no effect of frequency (r = −0.15, *p* = 0.90). The decreasing DPOAE level with increasing PTT reflected decreasing compressive nonlinearity with increasing SNHL up to the theoretical limit of active cochlear amplification (≤60 dB HL).

### 3.4. Psychophysical Tuning Curve (PTC)

Seven subjects (1, 2, 4–6, 8, and 10) were able to participate in the determination of PTC. The non-participating subjects found the test too demanding.

A variety of PTC-patterns existed. Four subjects (4, 5, 8, and 10) demonstrated a local minimum at the probe frequency (2000 Hz). Only subject 5 exhibited a normal PTC-pattern with a low-frequency ‘tail’ and a steep slope of the PTC-curve above the probe frequency (see Figure S1). Decreasing masking level with decreasing masking frequency was observed in subjects 8 and 10 at frequencies below the probe, i.e., they exhibited very high susceptibility for the upward spread of masking.

Subject 2 showed a minimum below the probe frequency, at 1560 Hz (the PTT was 20 dB better at 1560 than 2000 Hz), while subject 6 did not show any minimum in the PTC-curve. Subject 1 was highly susceptible to the upward spread of masking.

In all, 5 of 7 subjects (71%) demonstrated an increased upward spread of masking.

### 3.5. Wideband Tympanogram and Acoustic Stapedius Reflex Threshold (WBART)

Tympanic peak pressure (TPP) was normal. The average TPP was −6.5 daPa at 1000 Hz (95% confidence interval: −14.4–1.4 daPa, n = 10; similar TPPs at 2000 and 4000 Hz). Median WBART at 1000, 2000, and 4000 Hz was 81.0 dB HL (73.5–98.5 dB HL, n = 10), 83.0 dB HL (68.0 dB HL—non-measurable threshold (NT), n = 8), and NT (46.5 dB HL—NT, n = 8), respectively (subject 7 did not want to participate due to a feeling of discomfort for loud sounds. This subject revealed a normal tympanogram at the otomicroscopic examination performed immediately before the hearing tests).

### 3.6. Click-Evoked Entire ABR RMS Amplitude

**Figure 5:**
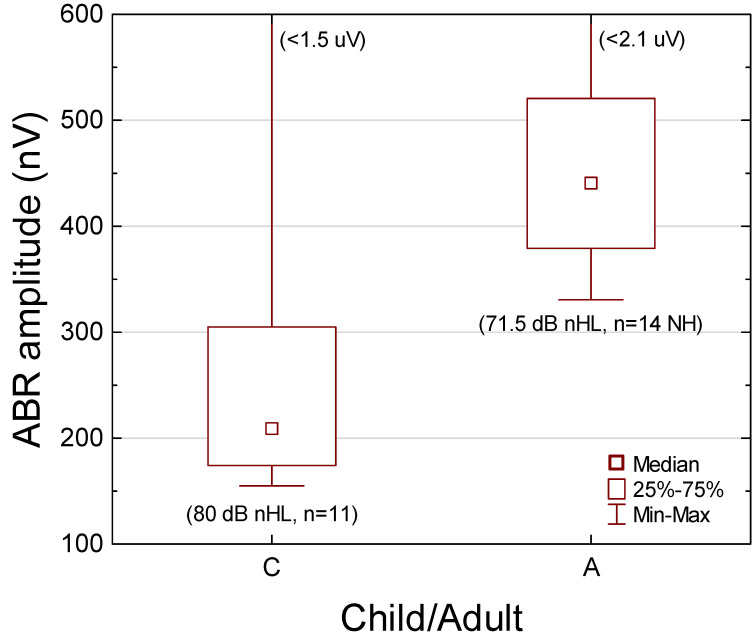
Comparison of click-evoked auditory brainstem response (ABR) RMS amplitudes (entire signal, ρ = 0.70) for the 11 hearing-impaired children (C, better ear) obtained at 80 dB nHL and 14 normal-hearing (NH) young adults (A, average between left and right ears) at 71.5 dB nHL [23]. Box-plots show median, quartiles, and span. Although the stimulus level was higher in the hearing-impaired children, the entire ABR RMS amplitude was significantly lower than in the NH young adults (*p* < 0.002, Mann–Whitney U test). Median ABR RMS amplitude in the children was 210 nV (at 80 dB nHL) and 440 nV in the adults (at 71.5 dB nHL).

Median ABR RMS amplitude at 80 dB nHL was 210 nV (155–1490 nV, QR 174–305 nV, n = 11; at ρ = 0.70) (left part in Figure 5). (Median ABR RMS amplitude for all the sweeps was similar; 216 nV, n = 11).

#### Comparison with Young Normal-Hearing Adults

Previously published ABR amplitudes for entire nonfiltered ABR in young NH adults (18–35 years of age) [23], as obtained in the same way as in the present study—but at a lower stimulus level (71.5 dB nHL)—demonstrated a median ABR RMS amplitude of 441 nV (331–2065 nV, QR 379–520 nV, n = 14; the average for left and right ears in each subject) (right part in Figure 5). Mann–Whitney U test revealed significantly different ABR RMS amplitudes (*p* < 0.002) between the HI children in the present study group (80 dB nHL, n = 11, better ear) and the NH adults (71.5 dB nHL, n = 14, average between the ears) [23] (Figure 5). Furthermore, the Kolmogorov–Smirnov test revealed significantly different cumulative distributions (*p* < 0.001).

According to [23], the slope of the ABR I/O function (ρ = 0.70) is 5.3 nV/dB. The extrapolated median amplitude is thus 486 nV for NH adults at 80 dB nHL (441 + 5.3∙8.5 = 486 nV), which was significantly different from that obtained in the HI children (*p* = 0.001, Mann–Whitney U test, n = 11 and 14, respectively). The ratio between the median ABR RMS amplitude for the HI children and the median estimated ABR RMS amplitude for the NH adults was 0.43 (210/486), as derived at equal stimulus levels. That is, the lower-than-normal suprathreshold ABR amplitudes should indicate a reduced number of firing neurons (see [65,66] for a quantitative relationship between surviving neurons and ABR amplitudes).

### 3.7. Click-Evoked ABR Wave Amplitudes and I-V Latency

Median ABR wave-I and wave-V amplitude at 80 dB nHL were 101 and 355 nV, respectively, while median ABR I-V latency was 4.10 ms (Table 2). Subject 3, who demonstrated the longest I-V latency (5.3 ms) and the lowest wave-V amplitude (71 nV), was the oldest subject at HA fit, 41 months.

No statistically significant relationship was found between ABR I-V latency (80 dB nHL at ≈11 years of age) and age at first HA-fit (r = 0.57, *p* = 0.10, n = 9; r = 0.03, *p* = 0.95, if the subject who had first HA-fit at 41 months of age was removed from the analysis). Hence, no evidence of persistently delayed maturation as a function of age at first HA-fit was demonstrated at the brainstem level for the subjects who received HAs typically at 6.0 months of age (QR 4–11 months, 4–41 months, n = 9).

#### Comparison with Young Normal-Hearing Adults

Mann–Whitney Utest revealed significant wave-I amplitude differences (*p* = 0.036) between the HI children (80 dB nHL, n = 10, better ear) and NH adults (71.5 dB nHL, unpublished results from [23], average between the ears, n = 12). Median ABR wave-I amplitude for the children was 101 nV (QR 50–162 nV, n = 10) and 186 nV (QR 99-225 nV, n = 12) for the NH adults. If the growth rate for ABR wave I is equal to the ABR RMS growth rate, the amplitude ratio is 0.44 (101/(186 + 5.3∙8.5)), as derived at equal stimulus levels. Using the estimated wave-I amplitudes for the young NH adults at 80 dB nHL, Mann-Whitney U-test revealed significant wave-I amplitude differences (*p* = 0.0056) between the HI children (n = 10) and the NH adults (n = 12).

Mann–Whitney U test did not reveal any significant wave-III amplitude differences (*p* = 1.0; 11 children, 14 adults), or, any significant wave-V amplitude differences (*p* = 0.46; 10 children, 14 adults) between the HI children (80 dB nHL) and the NH adults (71.5 dB nHL, unpublished results from [23]).

### 3.8. Click-Evoked ABR Threshold

Median ABRthr was 50 (QR 30–70, 20–80) dB nHL. To study the relationship between ABRthr and psychoacoustic thresholds, regression analyses were performed for PTTs between 2000–4000 Hz, i.e., for PTTs that correlate best to click-evoked ABRthrs [67,68]. A close relationship was found between the objectively defined ABRthrs and PTTs (2000–4000 Hz). The correlation coefficient between ABRthr and PTT at 2000, 3000, and 4000 Hz was 0.66, 0.80, and 0.78, respectively. At 3000 Hz, the regression line was ABRthr (dB nHL) = 3.2 + 0.90∙PTT (dB HL) (r = 0.80, *p* = 0.003, n = 11). The PTT averaged 50 dB HL at 2000–4000 Hz (Table S1, better ear), i.e., numerically equal to the ABRthr (mean and median ABRthr = 50 dB nHL, n = 11).

### 3.9. Aided Frequency-Modulated Tone Threshold in Sound Field

The median (QR) aided sound field threshold (FMT) as a function of frequency was relatively flat; 21.6(6.4), 18.6(3.6), 25.5(12.0), 29.0(19.4), 23.7(25.2), and 20.9(32.0) dB HL at 500–6000 Hz (n = 8 subjects). The overall aided median(mean) FMT was 22.6(23.2) dB HL (median across frequencies for the median as a function of frequency), thereby allowing the audibility of faint sounds. Subjects 4 and 5 did not use HAs (PTT < 30 dB HL in both ears at 2000 Hz, see Figure 2), and subject 6 did not bring HAs to the study day. All 8 subjects had non-linear HAs (Oticon (480 Vigo Pro, Safari 600, and Safari 900), Phonak Versata (3 subjects), and Widex AK 19), 5 of them with (active) gain controls.

### 3.10. Age at First Hearing Aid Fit and Daily Usage Time

The median age at the start of HA usage was 6.5 months (4–41 months, QR 4–11 months, n = 10). The mean (SD) daily HA usage time was 7.1(4.6) h (1–14 h, n = 9).

### 3.11. Recognition of Speech in Spatially Separate Competing Speech

Mean (median) unaided and aided SCS was −1.5 (−2.9) dB (n = 10) and −4.5 (−3.6) dB (n = 7), respectively, while the mean(median) SCS improvement was 5.3(4.5) dB (−3.8–18.4; Wilcoxon matched pairs test, *p* = 0.06, n = 7). Two subjects (4, 5) did not use HAs (PTTs < 30 dB HL at 2000 Hz, Figure 2), one subject did not bring the HAs to the test session, and one subject had too poor expressive language to participate in the test. Only subject 2 (12 years of age), who told the examiner that she did not use the HAs in noisy surroundings, revealed worsening aided SCS. She had aided FMTs ≤ 18 dB HL (500–6000 Hz). If subject 2 was excluded from the analysis, the aided SCS improvement was statistically significant (Wilcoxon matched pairs test: *p* < 0.03, n = 6; mean(median) aided improvement = 6.8(5.5) dB).

As a comparison, mean(SD) SCS in NH adults is −15.1(1.6) dB [69]. The effect of age up to approximately 14 years of age is −0.6 dB per year for NH children (linear regression analysis; Berninger, unpublished results, n = 48).

**Figure 6:**
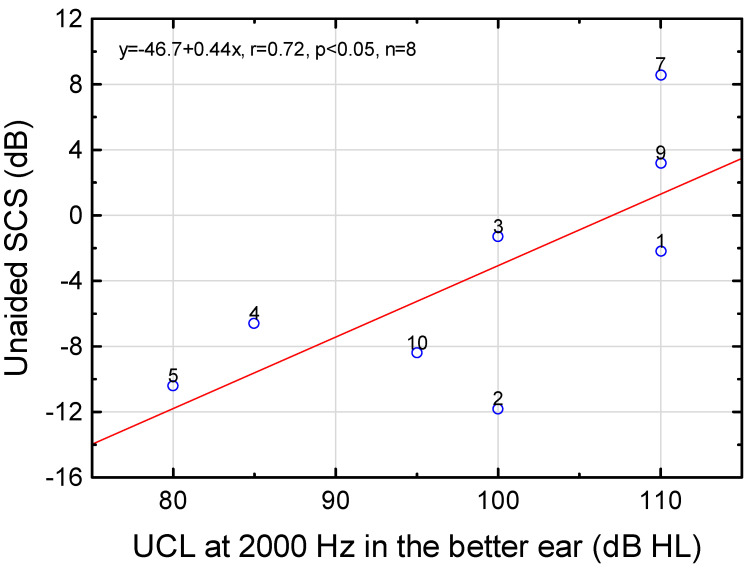
Unaided recognition of speech in competing speech (SCS) as a function of uncomfortable loudness level for pure tones (UCL) at 2000 Hz in the better ear for all the subjects who revealed UCLs within the measurable range (r = 0.72, *p* = 0.046, n = 8).

The unaided SCS was found to deteriorate with increasing UCL at 2000 Hz in the better ear (Figure 6). If the two subjects (6, 11) who exhibited non-measurable UCLs were included in the analysis (UCL was set to 130 dB HL), unaided SCS (dB) = −41.0 + 0.38∙UCL (dB HL) (r = 0.68, *p* = 0.029, n = 10).

Although the unaided SCS (but not UCL) was dependent on PTT at 2000 Hz (*p* = 0.006, r = 0.80, n = 10), i.e., audibility, the aided SCS did not depend on aided hearing threshold (r = 0.25, *p* = 0.58, n = 7). Hence, the major contribution to reduced SCS should not be audibility (see [70] for a discussion on audibility and distortion).

Given the significance of 2000–3000 Hz for speech recognition in noise [71,72], post-hoc analyses of unaided SCS grouped by normal or elevated UCLs in the better ear (elevated UCLs defined as ≥110 dB HL), at either 2000 or 3000 Hz, revealed statistically significant differences between the groups (Mann–Whitney U test, *p*’s = 0.032; exact p two-sided). The median SCS was −8.4 dB for the subjects with normal UCLs (n = 5) and 3.2 dB for elevated UCLs (n = 5; subjects 1, 6–7, 9, 11, see Figure 2 and Figure 6), i.e., a difference between the medians of 11.6 dB. The reduction of afferent signals was plausibly linked to worse SCS.

### 3.12. Questionnaires

Ten subjects participated in the APHAB questionnaire. Subject 8 did not participate due to poor expressive language. APHAB results were computed only for those subjects who fulfilled the required number of answers in both the unaided and aided conditions (as in [63]). As subjects 4 and 5 only had unaided APHAB (they did not use HAs) and subject 9 only had aided APHAB (HAs were used always), 7 subjects remained for the APHAB analyses.

The mean unaided(aided) percent of problems in the various listening categories “ease of communication” (EC), “reverberation” (RV), “background noise” (BN), and “aversiveness of sounds” (AV) was 43.8(24.3), 36.0(26.5), 54.0(44.5), and 24.5(36.6), respectively. (In comparison, first-time adult HA users revealed on average about 45(23), 50–60(25–30), 60(30–35), and 25(35–55) unaided(aided) percent of problems, respectively (better values for a digital HA), see [63]). Aided EC percent of problems was significantly lower than unaided EC (Wilcoxon matched pairs test, *p* < 0.02, n = 7), whereas aided AV percent of problems was significantly higher than unaided AV (Wilcoxon matched pairs test, *p* = 0.04, n = 7). Wilcoxon matched pairs test did not reveal any significant aided benefit in reverberant (RV; *p* = 0.4, n = 4) or noisy listening situations (BN; *p* = 0.2, n = 7).

Unaided percent of problems in EC (ECUA) was significantly related to PTA in the better ear: ECUA (%) = −8.4 + 1.3∙PTA (dB HL), (r = 0.95, *p* = 0.001, n = 7). That is, the lower PTA the lower the reported percent of problems in one-to-one communication. No significant relationship was found for RV (n = 4), BN (n = 7), or AV (n = 7).

Aided percent of problems in EC and BN were significantly related to aided sound field FMT average (FMTA, centre frequencies 500–2000 Hz): Aided EC (%) = −19.2 + 2.0∙FMTA (dB HL), (r = 0.87, *p* = 0.02, n = 6); Aided BN (%) = −31.5 + 3.7∙FMTA (dB HL), (r = 0.84, *p* = 0.04, n = 6). The lower FMTA, the lower the reported percent of problems in ease of communication and speech recognition in background noise.

A weak, although not statistically significant, relationship was found between aided benefit in BN and HA wearer’s usage time, as logged in each HA (r = 0.74, *p* = 0.056, n = 7); aided benefit in BN (% units) = −8.9 + 2.5∙Usage Time (h). Hence, the reported percent of problems for speech recognition in background noise tended to decrease with increased HA wearer usage time. Aided benefit in EC, RV, BN, and AV did not depend on age at first HA fit.

### 3.13. Imaging

Eight subjects completed the MRI. Subjects 6 and 9 did not show up despite two calls, and subject 11 could not relax during the MRI, hence the testing was terminated and this subject declined another MRI.

All the MRIs were normal in both ears (n = 8; subjects 1–5, 7–8, and 10). The MRI revealed normal inner ear structures in all the subjects, with clear imaging of the cochlea, the vestibular window, and the cochlear windows. The IAC and the cochlear and vestibular nerves revealed normal development. No abnormal radiological changes were found in the cochlea, vestibule, or IAC. No malformation of the inner ear structures, the cochlear, or the vestibular nerve was observed. No tumor pathology was identified.

The median time that elapsed between audiological testing and MRI was 111 days (QR 80–212 days, 27–696 days, n = 8).

## 4. Discussion

The main outcomes were quite symmetrical sigmoid SNHLs characterized by recruitment of loudness, increased upward spread of masking, statistically significant (minor) lateral asymmetries in PTT (right better than the left ear), DPOAEs more frequent than TEOAEs, DPOAE dependent on PTT (≤60 dB HL), distinctly reduced ABR RMS and wave-I amplitudes compared to young NH adults, and normal MRIs (MRI completed in 8 subjects). Unaided SCS was found to deteriorate with increasing UCL at 2000 Hz. The HAs fitted at the median age of 6.5 months allowed listening to soft sounds with a daily average HA use of 7 h. Most subjects demonstrated HA-benefit in a demanding listening situation with multiple spatially separate competing speech. Questionnaires revealed a weak (*p* = 0.056) relationship between aided benefit in BN and HA wearer daily usage time.

### 4.1. Hearing Dynamic Range

The reduced hearing dynamic range reflected the recruitment of loudness. The median PTT of 50 dB HL at 2000–4000 Hz (as recorded at ≈11 years of age) was similar to the median ABRthr at birth, i.e., 50 dB nHL. This similarity might reflect stable hearing thresholds—on a group level—if the neural maturation, which affects hearing sensitivity [13,14] in a frequency-dependent way at postsynaptic levels [73], outbalances the acoustical effect of the growth of the outer ear canal [12].

Minor HIs (>15 dB HL) should also be detected to avoid detrimental speech and language development [74,75], which is in line with our UNHS-criterion for normal ABR thresholds, 20 dB nHL [11]. This study revealed four subjects with PTAs 20–30 dB HL in the better ear (Figure 2). Two of them still used HAs at ≈11 years of age. As an example of the effect of a minor HI, children with unilateral, congenital, SNHL, who receive HAs late, demonstrate deteriorating aided horizontal sound localization accuracy in relation to increasing auditory nerve conduction time, which might reflect delayed maturation at the level of the brainstem [57]. In all, UNHS and early intervention programs that could detect minor SNHLs and a large incidence of congenital SNHL (e.g., [11,16]), should be a foundation for normal, or near-normal, maturation of the auditory pathways in young children with minor SNHL.

### 4.2. Effects of Ear and Sex on Hearing Thresholds

Statistically significant lateral asymmetries were found in the precisely recorded PTTs (SD for a single PTT ≤ 0.9 dB). Although the effect of sex did not reach significance (N.B. only four females), the median PTA was better in females than males. These results support earlier findings on the highly significant effects of ear and sex in normal neonatal TEOAE levels [15,76]. Significant lateral asymmetries and effects of sex also have been demonstrated for neonatal TEOAE pass ratios (right ear and females revealed higher pass ratios) along with a numerically higher proportion of SNHL in left ears and males [11], thus resembling pathophysiological differences, at birth. According to the hypothesis proposed by McFadden [26], and further studied by McFadden and colleagues (e.g., [77,78,79]), the effects of ear and sex may be due to prenatal masculinization processes and asymmetrical behavior of the efferent medial olivocochlear system. Efferent inhibition is relatively less in the right ears and females than in the left ears and males. However, evidence for the masculinization effect has not been found in a recent study on a large sample of neonatal twin TEOAEs [76]. Hence, more research is needed to elucidate the underlying mechanisms.

Better right than left ear PTTs have previously been demonstrated in non-noise-exposed young adults [80]. Here, we show statistically better right than left ear PTTs even for children with congenital SNHL.

### 4.3. Function of the Cochlear Amplifier

Only two subjects revealed TEOAEs (at 87 dB SPLp.e. ≈47 dB nHL [38]), whereas nine of the eleven subjects demonstrated positive DPOAE SNRs at any frequency (at 75 dB SPL). The DPOAEs were recordable at a higher median PTT (44 dB HL) than the TEOAEs (25 dB HL). According to the mammalian taxonomy proposed by [30], TEOAEs evoked at low stimulus levels are generated by coherent reflection, whereas DPOAEs are generated by non-linear distortion and linear reflection at the 2f1-f2 tonotopic place. More frequent DPOAEs than TEOAEs might, in part, be explained by the higher stimulus level used for the DPOAEs, which should be viewed in relation to the median PTT function ranging from 35–60 dB HL at 1000–6000 Hz (Figure 1; normal UCLs, see [64]). TEOAEs and DPOAEs evoked at relatively high stimulus levels add important clinical information on the function of the cochlear amplifier, such as sensitivity, compressive nonlinearity, and frequency resolution (see e.g., [19]). These OAEs are indicative of the degree of recruitment, which is important for gain-compression, maximum power output, and filter settings in the HAs.

The DPOAE level decreased by ≈4 dB for each 10 dB increase in PTT up to 60 dB HL (Figure 4, Table 1), which was supported by linear mixed modeling with PTT (*p* = 1∙10^−6^) and frequency (*p* = 0.90) as fixed effects. A decrease in the active compressive nonlinearity of the cochlea (e.g., [18,19]), as assessed by the DPOAEs, seems thus to be associated with increased PTTs in children with congenital, nonsyndromic, bilateral SNHL ≤ 60 dB HL. To our knowledge, this is a new finding that deserves future research for early diagnostics and HA fitting in infants. Another study gives some support to the present finding [81]. The reported absolute differences between extrapolated DPOAE thresholds and behavioral thresholds were ≤20 dB for children with cochlear hearing loss aged 4–15 years. The present material of congenital, alleged nonsyndromic, SNHL in children, and the use of high equal-level sinusoids for evoking DPOAEs might be important factors for the obtained relationship between the DPOAE and PTT. The mechanisms behind this potentially clinically important relationship remain to be studied.

### 4.4. Main Sites for Functional Deficiency

The statistically significant relationship between unaided SCS and UCL (2000 Hz), in combination with UCL not significantly dependent on PTT, and aided SCS not related to aided threshold, yet normal MRIs and normal average ABR wave I-V latency may reflect reduced SCS caused by, for example, a slight dysfunction at the level of the IHCs, the cochlear synapses or the endocochlear potential (cf. [82]). The suprathreshold (80 dB nHL) ABR wave-I and RMS amplitudes, which were 0.43–0.44 compared to young NH adults, corroborate this finding. In comparison, noise-exposed animals [49] and humans [83] reveal 40–60% lower suprathreshold wave-I amplitudes than controls, which may be attributed to cochlear synaptopathy. However, diverging results have been found in noise-exposed humans, in contrast to clear-cut results in animal models (see [84] for a review). In an experimental study in the rat, a close relationship between the electrically click-evoked ABR amplitude and the number of surviving spiral ganglion neurons has been shown [65,66], thereby emphasizing the importance of suprathreshold ABR measurements.

The click-evoked ABR-amplitude comparison between children at ≈11 years of age and adults, should be appropriate, as amplitude-intensity [54] and loudness functions [55] are similar. In addition, there is no change in composite click-evoked ABR amplitude (data collapsed over peaks I to VI) between five years of age and adulthood [56].

Interestingly, Anderson and Wedenberg [85] found a high prevalence of elevated ARTs in normal hearing carriers of genes for HI that could not be ascribed to a simultaneous elevation of PTTs; 63% in the hereditary series showed elevated ARTs, in contrast to only 3% in the control group. Recruitment of loudness, absent TEOAE in UNHS, reduced DPOAE as a function of PTT, reduced frequency selectivity, normal WBART (1000–2000 Hz) [86], and normal MRIs are pointing towards the OHCs and the cochlear amplifier as important sites for the unveiled functional deficiencies (see [87] for a pure OHC-based hearing loss). Future comprehensive genetic testing of all known deafness genes [88] would be important to achieve a link between (individual) auditory profiles and site-of-lesion, and above all, to the molecular level. High-level OAEs should be useful to achieve important clinical information on compressive nonlinearity which is important for HA fitting.

### 4.5. Hearing Aid Benefit

The mean aided benefit in SCS revealed a significant benefit in a demanding laboratory listening situation with multiple spatially separate interfering speech that should reflect a common and hard-to-master real-life listening situation. For those subjects who used HAs in noisy surroundings, the mean benefit was 6.8 dB, which should be viewed in the light of approximately 10%/dB slope of the psychometric function around 50% speech recognition. In comparison, a human-experimental study on temporary induced unilateral hearing loss in NH adults (43 dB average sound attenuation between 500–4000 Hz) showed a 3.0 dB mean reduction in SCS [69]. For first-time adult HA users, the mean aided benefit in SCS is 2.5 dB [63], which is considerably lower than the mean benefit demonstrated in this study.

A weak relationship, albeit not statistically significant (*p* = 0.056), between aided benefit in BN and HA wearer usage time (r = 0.74, n = 7) gives some support to the importance of early intervention and frequent use of HAs (7 h daily average HA use) for the development of binaural processing skills. A plausible explanation for this relationship is that the sensory environment shapes the brain functions during early sensitive periods [89,90,91] on the basis of frequent and adequate auditory stimulation for the development of auditory synapses [92].

The aided audibility for soft sounds should be important to enrich life and for awareness of the listening environment. The unaided percent of problems in EC was significantly related to unaided PTA, reflecting the importance of audibility in one-to-one communication. The aided EC percent of problems was significantly lower than unaided EC, whereas the aided AV percent of problems was significantly higher than unaided AV (e.g., traffic noise and sound of running water). An aided increase in AV has been shown for a variety of HAs in first-time adult HA users [63], thereby indicating a general problem using HAs in AV listening situations.

Interestingly, two of four subjects (2 and 10) with minor SNHLs, i.e., PTAs of 20–30 dB HL used HAs (three of these four subjects were fitted with HAs, i.e., two of the three subjects still used HAs, cf. Figure 2), thereby reflecting the importance to also detect minor SNHLs. In all, the study showed unambiguously HA-benefit.

### 4.6. Strengths and Limitations

The strengths of this study are the prospective cross-sectional design, the comprehensive test battery, and the well-defined material. The material is based on >30,000 consecutively screened newborns over a 6-year period (98% coverage rate in the UNHS), thereby reducing sample bias. Distinct inclusion criteria have been used. Hence, the material should constitute a representative sample of congenital, alleged nonsyndromic, bilateral SNHL identified at birth. Well-defined and highly accurate methods, the use of one and the same insert earphones throughout the study, randomized testing order, and all the audiological measurements performed in one audiometric test room with low ambient sound levels should ensure valid results. Supplementary questionnaires that were used to reflect self-reported HA benefit in real-life listening situations corroborated objective outcomes. Image technology, i.e., MRI, was included in the cross-sectional design to enhance detailed diagnostics by morphological studies of the cochlea and the IAC.

One limitation of generalization is the small sample size, however, if the used sample is representative, group data could be used to characterize nonprofound congenital SNHL, which is important for future clinical guidelines. Another limitation is that one of the subjects did not bring HAs to the test session and another subject had too poor expressive language to participate in the SCS test. None of the eight subjects who participated in the MRI displayed any anomaly. However, three subjects did not like to, or could not, complete or participate in the MRI, which limits the conclusions possible to draw from the MRI. (For a completely different study group, the congenital unilateral SNHL as identified at birth, MRI revealed malformations in 64% of all scans [93].)

### 4.7. Future Research and Clinical Implications

An unequivocal etiology should be paramount for optimizing the care of each child with congenital SNHL. The auditory function of the children in this study was assessed by electrophysiological, acoustical, and behavioral tests along with an MRI, i.e., test options available in most clinics. The fitting of HAs (and CIs) on the basis of a clear-cut etiology and detailed characteristics of the auditory function is an individualized intervention approach that should be studied extensively prior to clinical implementation. For example, reduced suprathreshold ABR amplitudes, elevated PTTs, and UCLs should require an alternative parameter setting in the HAs than for subjects exhibiting a pure OHC-based SNHL. Increased sensitivity to the upward spread of masking and reduced frequency selectivity typical for OHC-based SNHL, may to some extent be alleviated by digital processing in the HAs to enhance spectral contrasts [43], while high-pass filtering probably would increase recognition of speech in listening situations with speech-like or broadband background noise. Furthermore, attenuation of loud sounds might be a way to minimize coding deficits for subjects with cochlear synaptopathy [48]. Cytomegalovirus may induce a disturbance at the level of the stria vascularis in the organ of Corti, thereby affecting the endocochlear potential [94], which should require more linear HA gain without slow-acting compression. Awaiting clinical specific diagnoses (including e.g., comprehensive genetic testing of all known deafness genes to reach the molecular level [88] and cytomegalovirus diagnostics at birth), the present functional characterization would be helpful, for example, when fitting HAs to newborns and infants with congenital, alleged nonsyndromic, SNHL.

## 5. Conclusions

The typical congenital, nonsyndromic, nonprofound, and bilateral SNHL revealed sigmoid-like hearing thresholds of 50–60 dB HL at ≥1500 Hz, recruitment of loudness, increased upward spread of masking, distinctly reduced ABR RMS and wave-I amplitudes compared to young NH adults, and normal MRIs. Within the study group, DPOAE was dependent on the pure-tone threshold (≤60 dB HL) and unaided SCS was found to deteriorate with increasing UCL at 2000 Hz. The HAs fitted at the median age of 6.5 months allowed listening to soft sounds (median across frequencies, 23 dB HL) with a daily average HA use of 7 h. The mean HA-benefit (SNR) in a demanding listening situation with multiple spatially separate competing speech for those who used HAs in noisy surroundings was significant at 6.8 dB.

Specifically;

The systematic, albeit minor, lateral asymmetries in hearing thresholds support earlier findings in neonatal TEOAE levels, TEOAE pass ratios, and the proportion of SNHL in left/right ears.

High reliability in the determination of hearing thresholds (SD ≤0.9 dB) can be achieved by using a computerized Békésy technique.

The DPOAE evoked at high stimulus levels broadens the range of SNHL for which the cochlear function (e.g., sensitivity, compressive nonlinearity, and frequency resolution) could be analyzed. The distinct relationship between DPOAE and pure-tone threshold, up to the theoretical limit of active cochlear amplification, remains to be elucidated.

ABR amplitude is a clinically feasible and objective measure of the number of firing neurons. The lower-than-normal ABR amplitude should be a topic for future research.

Even minor SNHL could be identified by UNHS based on multiple recordings of TEOAEs and clinical brainstem response audiometry.

Even subjects with minor SNHLs (PTAs 20–30 dB HL) still used HAs at ≈11 years of age, reflecting the importance of also detecting minor SNHLs, at birth.

The significant relation between UCL and SCS has implications for HA-fitting, e.g., for gain-compression, maximum power output, and filter settings in the HAs. The reduction of afferent signals was plausibly linked to worse speech recognition in noise.

The fitting of HAs based on causes, mechanisms, and the functional characterization of the SNHL, may be an individualized intervention approach and deserves future research.

## Figures and Tables

**Table 1:** Regression analyses of 2f1-f2 distortion product otoacoustic emission (DPOAE) level (SNR ≥ 0 dB; left part) and 2f1-f2 DPOAE SNR vs. pure-tone threshold (PTT; right part) at various frequencies (PTT ≤ 60 dB HL).

Frequency (Hz)	Regression Line	r	*p*	n	Regression Line	r	*p*	n
1000	y = 13.1 − 0.25x	−0.49	0.18	9	y = 20.0 − 0.26x	−0.47	0.20	9
1500	y = 16.6 − 0.39x	−**0.80**	**0.05**	6	y = 31.2 − 0.45x	−0.80	0.06	6
2000	y = 23.9 − 0.70x	−**0.95**	**0.01**	5	y = 31.9 − 0.61x	−**0.96**	**0.01**	5
3000	y = 16.3 − 0.37x	−0.68	0.13	6	y = 31.1 − 0.37x	−0.65	0.16	6
4000	y = 16.8 − 0.40x	−**0.85**	**0.03**	6	y = 33.0 − 0.48x	−**0.90**	**0.01**	6
6000	y = 31.7 − 0.74x	−**0.95**	**0.01**	5	y = 49.8 − 0.83x	−**0.97**	**0.01**	5

DPOAE level (dB SPL); DPOAE SNR (dB); PTT (dB HL).

**Table 2:** Click-evoked auditory brainstem response amplitudes and wave I–V latency at 80 dB nHL (n = 10, except for wave III, n = 11).

	Min	Lower Quartile	Median	Upper Quartile	Max
Amplitude wave I	32	50	**101**	162	207
Amplitude wave III	36	169	**235**	407	462
Amplitude wave V	71	255	**355**	431	570
Latency I–V	3.38	3.82	**4.10**	4.20	5.26

Amplitude (nV); Latency (ms).

## Data Availability

All data are sharable upon request.

## Supplementary Materials

The following supporting information can be downloaded at: https://www.mdpi.com/article/10.3390/audiolres12050054/s1, Figure S1: Psychophysical tuning curve (PTC) in subject 5 who exhibited a quite normal PTC-pattern with a low-frequency ‘tail’ and a steep slope of the PTC-curve above the probe frequency (2000 Hz); Table S1: Mean (SD, range) and median pure-tone thresholds (dB HL) for left, right, better, and worse ear, respectively.

## Appendix A. Materials and Methods

### Appendix A.1. Subjects

#### Eligible Subjects

The median age for the 18 subjects at conclusive auditory brainstem response threshold (ABRthr) was 1.9 months (1.0–10.5 months), while the median age at SNHL determination was 53 months (25–83 months). Median ABRthr at birth for the left and right ears was 50 dB nHL (35 dB nHL—no response; quartile range (QR) 40–60 dB nHL), and 50 dB nHL (35 dB nHL—no response; QR 40–55 dB nHL), respectively. All but one of the 18 subjects used HAs in both ears. Subjects no. 1, 2, 5, 27, 28, 34, and 42 from Table I in [11] declined to participate in the study. For example, two of them had moved, one had attention deficit hyperactivity disorder (ADHD) (the parents had profound hearing loss), one subject had pending ADHD (this subject is studying in a special school), one subject had parents with profound hearing loss, one child would not participate (while parents were positive), and the remaining child was not interested in participating in the study.

### Appendix A.2. Study Design

Study day (forenoon): information, clinical examination including standard medical test protocol, otomicroscopy, tympanometry, and stapedius reflex measurements, pure-tone threshold (PTT), uncomfortable loudness level for pure tones (UCL), transient evoked otoacoustic emission (TEOAE), distortion product otoacoustic emission (DPOAE), psychophysical tuning curve (PTC) (30, 6∙2, 6∙2, 3, 3, 8 = 68 min); break; click-evoked ABR, wideband acoustic stapedius reflex threshold (WBART) (60, 6 = 66 min). Lunch.

Study day (afternoon): unaided speech in spatially separate competing speech (SCS), aided sound field threshold, aided SCS, questionnaires (15, 6, 15, 20 = 56 min).

### Appendix A.3. Hearing Dynamic Range for Pure Tones

#### Pure-Tone Threshold (PTT)

The PTT recording was first performed at 1000 Hz, then upward to 8000 Hz, followed by a retest at 1000 Hz, after which the PTT recording proceeded downward to 125 Hz.

Reliability in the PTT determination for a single measurement was calculated as *variance(test-retest)/2*, as *variance(test)* is assumed to be equal to *variance(retest)* and *variance(test-retest) = variance(test) + variance(retest)*.
