# Supplementary material for: Congenital Nonprofound Bilateral Sensorineural Hearing Loss in Children: Comprehensive Characterization of Auditory Function and Hearing Aid Benefit
Source: Audiol Res. 2022 Oct 7;12(5):539–63. doi: 10.3390/audiolres12050054 (PMC9598400; doi:10.3390/audiolres12050054)
Supplement: Supplementary file 1 [file audiolres-12-00054-s001.zip › Table S1.pdf]

Table S1. Mean (SD, range) and median pure-tone thresholds (dB HL) for left, right, better, and worse ear, respectively (n=11, except at 6000 and 8000 Hz. * denotes n=10))

		Pure-tone threshold (dB HL)			
Frequency		Left ear	Right ear	Better ear	Worse ear
125 Hz	Mean	25.0	24.8	24.1	25.7
	SD	15.6	19.2	19.0	15.8
	Median	17.4	20.9	22.4	17.4
	Min	9.0	6.6	6.6	9.0
	Max	53.4	71.5	71.5	53.4
250 Hz	Mean	26.8	24.7	24.4	27.0
	SD	17.5	15.7	14.7	18.3
	Median	19.2	21.8	21.8	19.2
	Min	8.6	9.9	10.0	8.6
	Max	70.1	57.6	57.6	70.1
500 Hz	Mean	33.8	31.6	31.0	34.4
	SD	20.4	21.3	19.9	21.6
	Median	28.9	24.6	24.6	28.9
	Min	10.7	8.1	10.7	8.1
	Max	80.6	81.4	81.4	80.6
1000 Hz	Mean	43.5	39.3	38.3	44.4
	SD	17.0	16.1	16.1	16.7
	Median	42.7	34.9	34.9	42.7

	Min	20.4		16.5	16.5	24.0	
	Max	83.5		72.3	72.3	83.5	
1500 Hz	Mean	50.6		42.3	47.2	45.7	
	SD	24.2		19.1	24.8	19.4	
	Median	47.5		43.8	47.5	43.8	
	Min	20.6		15.5	15.5	20.6	
	Max	93.2		78.2	93.2	80.7	
2000 Hz	Mean	53.5		49.3	50.0	52.8	
	SD	19.7		17.8	18.4	19.3	
	Median	56.1		48.1	46.3	54.5	
	Min	25.4		19.8	19.8	25.4	
	Max	85.2		73.3	73.3	85.2	
3000 Hz	Mean	54.4		49.4	49.2	54.6	
	SD	20.8		17.6	17.8	20.5	
	Median	55.2		49.2	46.9	54.5	
	Min	23.9		19.3	19.3	23.9	
	Max	101.1		79.8	79.8	101.1	
4000 Hz	Mean	54.2		52.8	52.2	54.8	
	SD	21.3		25.5	25.5	21.2	
	Median	54.5		52.3	48.0	53.7	
	Min	13.4		7.0	7.0	13.4	
	Max	99.1		106.5	106.5	99.1	
6000 Hz	Mean	56.0	*	59.9	60.9	55.0	*
	SD	19.8		20.8	21.0	19.3	

	Median	58.3	58.7	60.0	58.3
	Min	12.1	22.6	22.6	12.1
	Max	81.0	95.6	95.6	79.0
	Mean	53.4 *	55.6 *	52.4 *	56.6 *
	SD	20.2	22.2	20.7	21.6
8000 Hz	Median	60.1	58.7	58.7	60.1
	Min	15.4	9.7	9.7	15.4
	Max	78.5	84.4	78.5	84.4
