# Supplementary figures and images for: Congenital Nonprofound Bilateral Sensorineural Hearing Loss in Children: Comprehensive Characterization of Auditory Function and Hearing Aid Benefit
Source: Audiol Res. 2022 Oct 7;12(5):539–63. doi: 10.3390/audiolres12050054 (PMC9598400; doi:10.3390/audiolres12050054)

## Slide 1
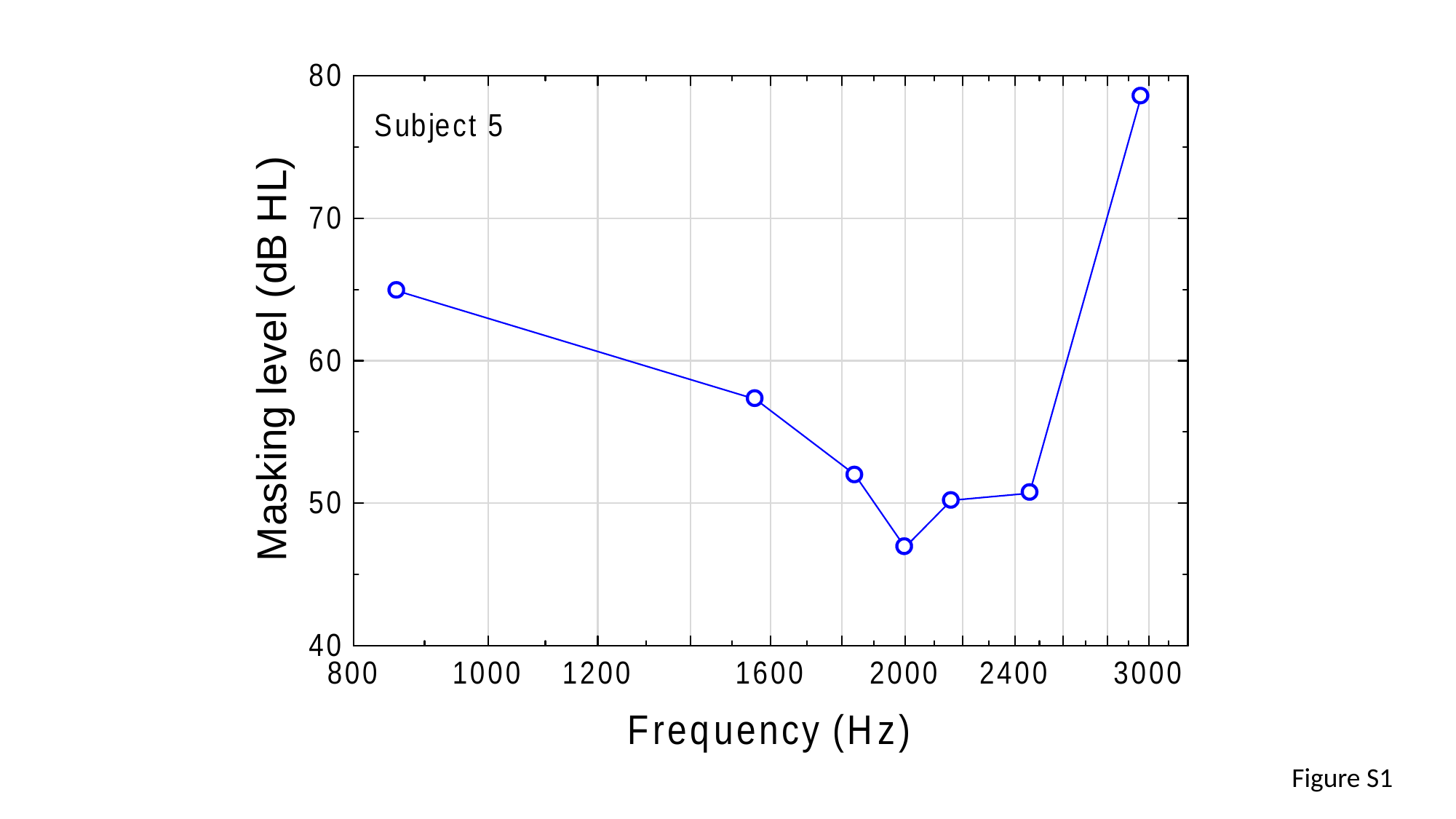

Figure S1

Supplement: Supplementary file 1 [file audiolres-12-00054-s001.zip › Figure S1.pptx]
